# Synthesis and Spectroscopic Properties of New Azo Dyes Derived from 3-Ethylthio-5-cyanomethyl-4-phenyl-1,2,4-triazole

**DOI:** 10.3390/molecules19032993

**Published:** 2014-03-07

**Authors:** Mariam Al-Sheikh, Hanadi Y. Medrasi, Kamal Usef Sadek, Ramadan Ahmed Mekheimer

**Affiliations:** 1 Department of Chemistry, Faculty of Science for Girls, King Abdulaziz University, Jeddah, P.O. Box 50918, Jeddah 21533, Saudi Arabia; 2 Department of Chemistry, Faculty of Science, El-Minia University, El-Minia 61519, Egypt

**Keywords:** synthesis, azo-hydrazone tautomerism, coupling reaction, diazotization

## Abstract

New 1,2,4-triazole colorants were obtained, in high yields, by coupling 3-ethylthio-5-cyanomethyl-4-phenyl-1,2,4-triazole (**1**) with diazotized aniline derivatives **2**, **4** and **6**. The azo dyes prepared in this work may exist in three tautomeric forms. We found that the tautomerism is influenced mainly by the nature of substituent at the *para* position of the aniline coupling component. This tautomerisation was observed in the NMR spectra of the dyes. The dyes were characterized by IR, ^1^H-NMR, ^13^C-NMR and MS spectroscopic techniques.

## 1. Introduction

Azo-functionalized dyes bearing aromatic heterocyclic components [1] have attracted ever increasing attention in recent years due to their wide range of color, brightness, simplicity and ease of manufacturing and good dyeing performance [2,3,4,5]. They are used in high tech applications such as lasers and non-linear optical systems [6], thermal transfer printing and fuel cells [7], dye sensitized solar cells [8], photodynamic therapy [9], and metallochromic indicators [10]. They are also used in dyeing textiles, leather, paper, food and cosmetic products [11]. Furthermore, azo dye compounds are known for their medicinal importance [12,13,14,15] and are also known to be involved in a number of biological reactions such as inhibition of DNA, RNA and protein synthesis, carcinogenesis and nitrogen fixation [16]. In a broader sense, the azo dyes constitute the largest diverse group of all the synthetic colorants [17]. In addition, hydrazones are the well known class of biologically and pharmacologically active compounds in the field of synthetic chemistry [18,19,20]. Some hydrazones have been introduced as potent drugs such as gyromitrin [21] used as a toxin and dihydralazine [22] used as a hypertensive drug. Moreover, hydrazones are an important class of chemical intermediates, which can act as electrophiles and as nucleophiles in chemical reactions [23,24,25,26,27]. 1,2,4-Triazoles and their derivatives play an important role in modern drug discovery and have attracted attention from both industrial and academic groups. These systems are important pharmaceuticals due to their interesting biological activities [28,29,30,31]. Several compounds containing 1,2,4-triazole rings are well known as drugs. For example, vorozole, letrozole, and anastrozole are non-steroidal drugs used for the treatment of cancer [32], while loreclezole is used as anticonvulsant [33] and fluconazole is used as an antimicrobial drug [34]. In the light of the above report and in continuation to our previous work on the synthesis of heterocyclic systems containing 1,2,4-triazole moiety [35,36,37], the present work focuses on the synthesis, spectroscopic properties of some novel azo dyes derived from 3-ethylthio-5-cyanomethyl-4-phenyl-1,2,4-triazole [35]. Furthermore, we also examined the effect of substituent at the *para* position of the aniline coupling component on the nature of the resulting products.

In solution, the azo dyes theoretically may be involved in azo-hydrazone tautomerism. Since the tautomeric ratio is important for the industrial application of azo dyes, determination of azo-hydrazone tautomerism (AHT) in the solid state and in solution is of interest both from a theoretical and practical aspects because the two tautomers have different technical properties and dyeing performance [38]. Therefore, it was considered worthwhile to determine the tautomeric structure of the products prior to exploring their applications.

## 2. Results and discussions

**Scheme 1:**
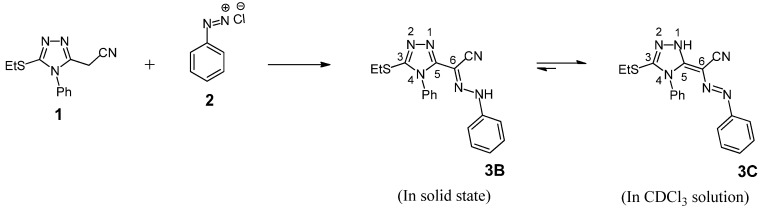
Coupling reaction of **1** with benzene diazonium chloride.

As a starting point for our investigation, we first examined the coupling reaction of compound **1** with benzenediazonium chloride (**2**). Thus, coupling of diazonium salt **2** with compound **1** in aqueous ethanol in the presence of a buffered sodium acetate solution gave 5-ethylthio-*N****'***,4-diphenyl-4*H*-1,2,4-triazole-3-carbohydrazonoyl cyanide (**3B**), as the only isolable product, in excellent yield (Scheme 1).

**Figure 1:**
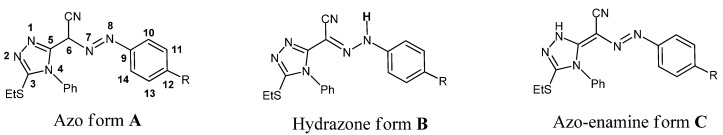
Three tautomeric structures of diazonium coupling product of **1**.

The prepared dye may exist in three possible tautomeric forms, namely the azo form **A**, the hydrazone form **B** and the azo-enamine form **C**, as depicted in Figure 1. The IR spectrum (in KBr) revealed the presence of absorption bands at *v* 3236 and 2213 cm^−1^ due to the NH and cyano groups, respectively. On the other hand, the other *v*_max_ value at 1231 cm^−1^ was assigned to the N-N stretching mode [39].

Kostyuchenko *et al*. reported that the molecular ion of tautomeric monoazo dyes cleaves preferentially at the N-N bond in the hydrazone and at one of the C-N bonds in the azo tautomer, yielding high abundance fragments with corresponding metastable ions [40]. In the mass spectrum of **3B**, the respective molecular ion peak (M^+^) and the (M^+^ + 1) peak were observed. In addition, the spectrum showed characteristic peaks at *m/z* values corresponding to C_6_H_5_NH (resulting from cleavage at the N-N bond), C_8_H_5_N_3_ and C_10_H_10_N_3_S ion fragments. The latter two fragments correspond to 4-phenyl-1,2,4-triazole and 3-ethylthio-4-phenyl-1,2,4-triazole residues, respectively. Moreover, the base peak that appeared at *m/z* 77 with relative intensity of 100% is due to cleavage of the phenylium cation (Ph^+^) from M^+^. Taken together the data is in good agreement with the proposed hydrazone structure. The ^1^H-NMR spectral data shows that two tautomeric forms **3B** (hydrazone form) and **3C** (azo-enamine form) are present in CDCl_3_ solution with relative intensities of 1:3 (Scheme 1, Table 1).

**Table 1:** Tautomer ratios in the solid state and in CDCl_3_ solution.

Product	In Solid State	In CDCl_3_ Solution
	Azo Hydrazone	Hydrazone:Azo-enamine
**3**	^___^ 100	**3B:3C** (25:75)
**5**	100 ^___^	**5B:5C** (20:80)
**7**	100 ^___^	**7B:7C** (60:40)
**8**	^___^ 100	**8B:8C** (17:83)

In the ^1^H-NMR spectrum a singlet at δ = 8.83 ppm [41] is due to N-H proton of hydrazone form **3B** (25%) and the other downfield singlet at δ = 13.71 ppm [42] was assigned to the triazole N-H in the azo-enamine form **3C** (75%). Tautomeric ratios were calculated from their ^1^H-NMR integrals by comparison of the NH signal of the hydrazone form **3B** and NH signal of the azo-enamine form **3C**. Therefore, ^1^H-NMR chemical shift data can readily be employed to study the tautomeric equilibria quantitatively. Also, the ^13^C-NMR spectrum of this product in CDCl_3_ displayed signals in agreement with the mixture of two tautomers, hydrazone form **3B** and azo-enamine form **3C**. The spectrum showed besides the signals due to aromatic, ethyl, cyano and triazole carbones, two characteristic signals at δ = 99.05 and 140.75 ppm attributable to the carbon atom at position 6 in both tautomeric forms **3C** and **3B**, respectively (see Experimental). Due to the novelty of this product, the ^13^C-NMR chemical shifts values were assigned for these carbon atoms by comparing the experimental data in the ^13^C-NMR spectrum of the product with the ^13^C-NMR chemical shifts of theoretical results for molecular modeling using ChemBio3D Ultra 12.0 [43].

**Scheme 2:**
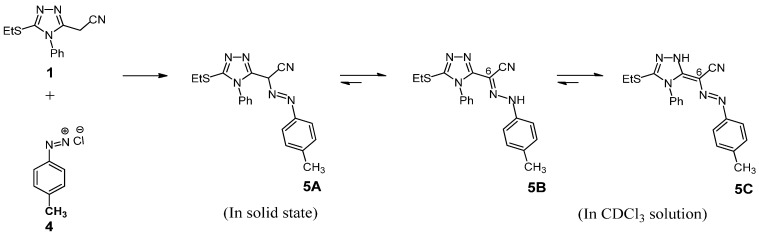
Coupling reaction of **1** with diazotized 4-methylaniline.

Next, we examined the effect of substitution at the *para-*position of the diazonium salt benzene ring on the equilibrium between the three forms **A**–**C** (Figure 1). Recently, Pavlović and his co-workers [44] have been reported that the electron-releasing substituents at the *para* position of the diazonium salt benzene ring increase the azo form content, while electron-withdrawing groups increase the content of the hydrazone form. In accordance with these results, it was found that the coupling reaction of **1** with diazotized 4-methylaniline (**4**), under similar reaction conditions as above, afforded 5-ethyl-thio-3-(1-(4-methylphenylazo)-4-phenyl-acetonitrile)-4*H*-1,2,4-triazole (**5A**), in 82% yield (Scheme 2).

The structure of this azo dye was verified by elemental analyses and spectroscopic methods (IR, MS, ^1^H- and ^13^C-NMR). Structure **5A** seemed to be logical according to the IR spectrum (in KBr) which disclosed no amino group (NH) absorption band and the presence of intense cyano and azo (‑N=N-) [45] bands at 2217 and 1547 cm^−1^, respectively. The mass spectral data of azo dye **5A** showed a molecular ion peak (M^+^) at *m/z* 362 (40%) which was in concordance with the molecular mass (362) of the product (C_19_H_18_N_6_S). In addition, cleavage at one of the C-N bonds in the azo tautomer **5A** led to the appearance of the base peak at *m/z* 91 (CH_3_-C_6_H_4_) with relative intensity of 100%. Moreover, the spectrum showed characteristic peaks at *m/z* 119 (14%), 143 (6%), 156 (11%) and 243 (4%) corresponding to CH_3_-C_6_H_4_-N=N (resulting from cleavage at the CN bond), C_8_H_5_N_3_ (4-phenyl-1,2,4-triazole), C_8_H_5_N_3_-CH (4-phenyl-1,2,4-triazole with CH group at C-3), and C_10_H_10_N_3_S-CH-CN (3-ethylthio-4-phenyl-1,2,4-triazole with CH-CN group at C-5) residues, respectively. This would suggest that compound **5A** exist almost exclusively in the azo form. Interestingly, the ^1^H-NMR spectral data shows that the hydrazone form **5B** and azo-enamine tautomeric form **5C** are present in CDCl_3_ solution with relative intensities of 1:4 (Scheme 2, Table 1). The ^1^H-NMR spectrum revealed a similar pattern as observed for **3B** and **3C** (see Experimental). Also, the spectrum do not show any more signals around 4.5 ppm which is usually reported for the methine proton of azo form **5A** of the annulated similar compounds [46]. In addition, the ^13^C-NMR spectrum of this product in CDCl_3_ showed signals in accordance with the mixture of two tautomers, hydrazone form **5B** and azo-enamine form **5C**. The spectrum showed besides the signals due to aromatic, ethyl, methyl, cyano and triazole carbones, two characteristic signals at δ = 98.34 and 139.57 ppm attributable to carbon atom at position 6 in both tautomeric forms **5C** and **5B**, respectively (see Experimental).

**Scheme 3:**
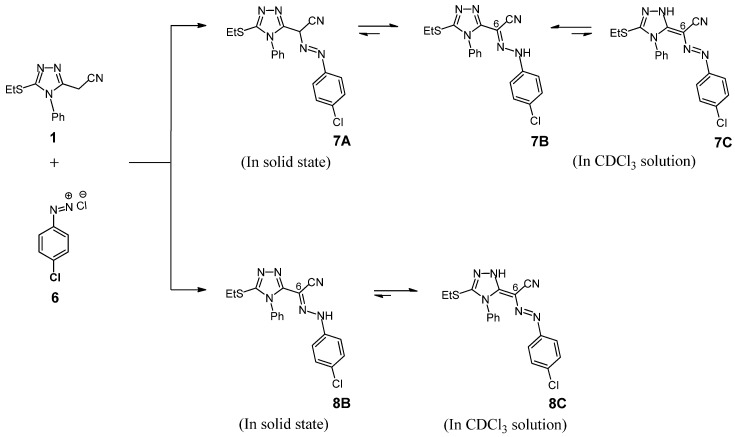
Coupling reaction of **1** with diazotized 4-chloroaniline.

Attention was next turned to investigate the coupling reaction of the diazonium salts having electron withdrawing substituents at *para* position of benzene ring with compound **1**. Surprisingly, when compound **1** was coupled with diazotized 4-chloroaniline (**6)**, under the same reaction conditions as above, it afforded two tautomers, **7A** (major product) and **8B** (minor product) (Scheme 3), which readily separated by preparative TLC (PLC) using silica gel.

To the best of our knowledge, this is the first reported isolation of two isomers in solid state in such reactions. The identity of major product **7A** was supported by spectroscopic data. For example, its mass spectrum showed a molecular formula C_1__8_H_15_ClN_6_S (M^+^ 382) and peaks at 139 (Cl-C_6_H_4_-N=N-, 17%) and 111 (Cl-C_6_H_4_, base peak, 100%) confirming its presumed structure (see Experimental). The IR spectrum showed no amino group (NH) absorption, but absorption bands for CN and -N=N- groups were observed at *v* = 2217 and 1547 cm^−1^, respectively. Interestingly, the ^1^H-NMR spectral data shows that the azo-enamine tautomeric form **7C** and hydrazone form **7B** are present in CDCl_3_ solution with relative intensities of 1:1.5 (Scheme 3, Table 1). The ^1^H-NMR spectrum disclosed, besides the characteristic signals for the ethyl and aromatic protons, only two singlet signals at δ = 8.91 and 13.74 ppm attributable to N-H proton of the hydrazone form **7B** and triazole N-H in the azo-enamine form **7C**, respectively. Also, the ^13^C-NMR spectrum of this product in CDCl_3_ displayed signals in agreement with the mixture of two tautomers, the hydrazone form **7B** and azo-enamine form **7C** (see Experimental). The structure of the minor product **8B** was fully confirmed with the help of analytical and spectroscopic data. Particularly, the IR spectrum showed an amino group (NH) absorption band. Moreover, its structure is supported by its mass spectrum which showed the molecular ion peak at *m/z* 382 (29%), which is consistent with its structural formula. Other prominent peaks that observed at *m/z* 126 (Cl-C_6_H_4_-NH, 12%) and 111 (Cl-C_6_H_4_, base peak, 100%) confirming its presumed structure (see Experimental). However, the ^1^H-NMR spectrum revealed two singlet signals for the N-H proton of the hydrazone form **8B** and the triazole N-H in the azo-enamine form **8C** with relative intensities of 1:5 (Table 1). This may be interpreted by assuming that the product **8B** exists in CDCl_3_ as a mixture of the two tautomeric forms **8B** and **8C** (cf. Scheme 3). Also, the ^13^C-NMR spectrum of this product in CDCl_3_ displayed signals in agreement with the mixture of two tautomers, the hydrazone form **8B** and the azo-enamine form **8C** (see Experimental). Unfortunately, we did not succeed in growing the single crystal of compounds **3**, **5**, **7** and **8** suitable for X-ray crystallographic analysis.

## 3. Experimental

### 3.1. General

Melting points were measured on a Gallenkamp apparatus and are not corrected. IR spectra (KBr) were recorded with a Nicolet Magna 520FT IR spectrophotometer. Peaks are reported in cm^−1^. ^1^H and ^13^C-NMR spectra were recorded on a Bruker DPX (600 MHz for ^1^H-NMR and 150 MHz for^ 13^C-NMR) spectrometer in CDCl_3_ using TMS as an internal standard; the chemical shifts are given in δ units (ppm). Mass spectra were performed on a Shimadzu GCMS-QP 1000 EX mass spectrometer at 70 eV. Analytical thin-layer chromatography (TLC) was performed on aluminum sheets precoated with silica gel (Merck, Kieselgel 60 PF_254_). Visualization was accomplished by UV light. Microanalytical data were obtained from the Microanalytical Data Unit at Cairo University, Egypt.

### 3.2. General Procedure for the Synthesis of Arylhydrazone (or Arylazo) Compounds **3**, **5**, **7** and **8**

A cold solution of aryldiazonium salt (4 mmol) was prepared by adding a sodium nitrite solution (0.4 g, 6 mmol, dissolved in 2 mL water) to a pre-cooled solution of arylamine hydrochloride (4 mmol of either of the appropriate aniline derivatives **2** and **4** in 2 mL of 6 M hydrochloric acid) with continuous stirring. The resulting solution of the aryl diazonium salt was then added carefully to a cold solution of 1,2,4-triazole derivative **1** (4 mmol) in ethanol (40 mL) containing sodium acetate (0.66 g in 2 mL H_2_O). The reaction mixture was stirred at room temperature for 24 h and the resulting solid product was collected by filtration, washed well with H_2_O and dried to afford compounds **3B** and **5A**, respectively, which were purified by preparative TLC using silica gel plates (toluene/acetone, 10:2), followed by recrystallization from EtOH. In the case of the reaction of **1** with **6**, the resulting solid product was chromatographed on a preparative TLC plate using 10:3 toluene/acetone as eluent to give two zones. Extraction with acetone followed by recrystallization from EtOH gave compounds **7A** and **8B**, respectively.

*2-(5-Ethylthio-4-phenyl-4H-[1,2,4]triazol-3-yl)-2-(phenylhydrazono)acetonitrile* (**3B**). Yellow crystals. Yield (1.28 g, 90%); m.p.: 172–174 °C. IR (KBr): *v* = 3236 (NH), 2937 (aliph. CH), 2213 (CN), 1594 (C=N), 1231 (N-N) cm^−1^; ^1^H-NMR: δ = 1.44 (t, 3H, *J* = 7.2 Hz, CH_3_), 1.48 (t, 3H, *J* = 7.2 Hz, CH_3_), 3.28–3.34 (m, 4H, 2 CH_2_), 6.38 (d, 2H, *J* = 8.4 Hz, ArH), 6.99 (t, 1H, *J* = 8.4 Hz, ArH), 7.12–7.15 (m, 5H, ArH), 7.31–7.32 (m, 2H, ArH), 7.35–7.42 (m, 6H, ArH), 7.59–7.63 (m, 3H, ArH), 7.65–7.68 (m, 1H, ArH), 8.83 (s, 0.25H, hydrazone NH), 13.71 (s, 0.75H, triazole NH); ^13^C-NMR: δ = 14.65 (CH_3_), 14.67 (CH_3_), 26.61 (CH_2_), 26.75 (CH_2_), 99.05 (=C-CN in azo-enamine form), 114.27 (CN), 114.30 (CN), 115.53 (3 Ar-C), 123.97 (1 Ar-C), 124.80 (2 Ar-C), 127.35 (1 Ar-C), 128.20 (3 Ar-C), 129.27 (1 Ar-C), 129.53 (3 Ar-C), 129.82 (1 Ar-C), 130.09 (3 Ar-C), 131.54 (2 Ar-C), 131.59 (1 Ar-C), 134.54 (1 Ar-C), 140.75 (CN-C=N-NH in hydrazone form), 141.81 (2 Ar-C), 148.47 (triazole C-3), 148.60 (triazole C-3), 154.14 (triazole C-5), 154.97 (triazole C-5); MS *m/z* (rel. int. %) 349 (M^+^ + 1, 10), 348 (M^+^, 38), 347 (22), 320 (9), 319 (7), 290 (4), 244 (3), 243 (4), 242 (6), 215 (9), 204 (2), 155 (6), 149 (6), 143 (3), 129 (6), 128 (5), 105 (19), 92 (6), 91 (8), 77 (100), 76 (67), 65 (20), 64 (14), 63 (7), 61 (6), 52 (5), 51 (26), 50 (14); Anal. Calcd. for C_18_H_16_N_6_S (348.42): C, 62.05; H, 4.63; N, 24.16; S, 9.20. Found: C, 61.91; H, 4.78; N, 24.35; S, 9.06.

*2-(5-Ethylthio-4-phenyl-4H-1,2,4-triazol-3-yl)-2-(4-methylphenyldiazenyl)acetonitrile* (**5A**). Yellow crystals. Yield (1.22 g, 82%); m.p.: 169–170 °C. IR (KBr): *v* = 2980, 2920 (aliph. CH), 2217 (CN), 1594 (C=N), 1547 (-N=N-) cm^−1^; ^1^H-NMR: δ = 1.43 (t, 3H, *J* = 7.2 Hz, CH_3_), 1.47 (t, 3H, *J* = 7.2 Hz, CH_3_), 2.26 (s, 3H, CH_3_), 2.34 (s, 3H, CH_3_), 3.28-3.34 (m, 4H, 2 CH_2_), 6.27 (d, 2H, *J* = 8.4 Hz, ArH), 6.94 (d, 2H, *J* = 8.4 Hz, ArH), 7.18 (d, 2H, *J* = 8.4 Hz, ArH), 7.30-32 (m, 5H, ArH), 7.35–7.37 (m, 3H, ArH), 7.59–7.63 (m, 3H, ArH), 7.65–7.67 (m, 1H, ArH), 8.78 (s, 0.20H, hydrazone NH); 13.70 (s, 0.80H, triazole NH); ^13^C-NMR: δ = 14.66 (CH_3_), 14.68 (CH_3_), 20.70 (CH_3_), 20.92 (CH_3_), 26.60 (CH_2_), 26.76 (CH_2_), 98.34 (=C-CN in azo-enamine form), 114.21 (CN), 114.50 (CN), 115.48 (4 Ar-C), 127.37 (1 Ar-C), 128.22 (3 Ar-C), 129.78 (2 Ar-C), 130.04 (2 Ar-C), 130.07 (4 Ar-C), 131.50 (2 Ar-C), 131.64 (1 Ar-C), 133.75 (1 Ar-C), 134.58 (1 Ar-C), 134.64 (2 Ar-C), 138.48 (1 Ar-C), 139.57 (CN-C=N-NH in hydrazone form), 148.55 (triazole C-3), 148.73 (triazole C-3), 153.97 (triazole C-5), 154.75 (triazole C-5); MS *m/z* (rel. int. %) 363 (M^+^ + 1, 11), 362 (M^+^, 40), 361 (21), 348 (5), 347 (6), 346 (8), 345 (6), 335 (6), 334 (16), 333 (10), 306 (9), 305 (11), 274 (4), 273 (5), 257 (5), 243 (4), 242 (6), 231 (4), 230 (5), 215 (14), 188 (4), 157 (8), 156 (11), 149 (11), 148 (7), 144 (6), 143 (6), 128 (8), 119 (14), 118 (13), 117 (9), 106 (8), 105 (22), 104 (13), 103 (11), 97 (6), 92 (14), 91 (100), 90 (26), 77 (49), 76 (17), 66 (6), 65 (27), 64 (21), 63 (12), 61 (6), 60 (9), 59 (13), 56 (9), 51 (26); Anal. Calcd. for C_19_H_18_N_6_S (362.45): C, 62.96; H, 5.01; N, 23.19; S, 8.85. Found: C, 63.14; H, 4.87; N, 23.30; S, 9.01.

*2-(4-Chlorophenyldiazenyl)-2-(5-ethylthio-4-phenyl-4H-1,2,4-triazol-3-yl)acetonitrile* (**7A**)*.* Yellow crystals. Yield (0.785 g, 50%); m.p.: 118–120 °C. IR (KBr): *v* = 2925 (aliph. CH), 2217 (CN), 1597 (C=N), 1547 (-N=N-) cm^−1^; ^1^H-NMR: δ = 1.43 (t, 3H, *J* = 7.2 Hz, CH_3_), 1.48 (t, 3H, *J* = 7.2 Hz, CH_3_), 3.27–3.35 (m, 4H, 2 CH_2_), 6.30 (d, 2H, *J* = 9 Hz, ArH), 7.10 (d, 2H, *J* = 9 Hz, ArH), 7.30–7.36 (m, 8H, ArH), 7.57–7.68 (m, 6H, ArH), 8.91 (s, 0.60H, hydrazone NH), 13.74 (s, 0.40H, triazole NH); ^13^C-NMR: δ = 14.65 (2 CH_3_), 26.62 (CH_2_), 26.76 (CH_2_), 99.68 (=C-CN in azo-enamine form), 108.93 (CN), 114.02 (CN), 115.46 (2 Ar-C), 116.65 (2 Ar-C), 127.33 (3 Ar-C), 128.17 (2 Ar-C), 128.99 (1 Ar-C), 129.28 (4 Ar-C), 129.61 (1 Ar-C), 129.85 (1 Ar-C), 130.12 (4 Ar-C), 131.48 (1 Ar-C), 131.61 (1 Ar-C), 134.54 (1 Ar-C), 139.48 (1 Ar-C), 140.44 (CN-C=N-NH in hydrazone form), 148.30 (triazole C-3), 148.49 (triazole C-3), 154.37 (triazole C-5), 155.11 (triazole C-5); MS *m/z* (rel. int. %) 384 (M^+^, 27), 382 (M^+^, 78), 381 (20), 356 (4), 355 (7), 354 (13), 353 (15), 352 (5), 328 (7), 327 (10), 326 (13), 325 (17), 324 (7), 293 (7), 244 (4), 243 (9), 242 (15), 241 (7), 232 (3), 231 (3), 215 (15), 214 (10), 213 (7), 192 (3), 191 (8), 183 (4), 182 (6), 181 (7), 167 (5), 157 (10), 156 (25), 155 (13), 149 (12), 148 (5), 143 (3), 142 (6), 141 (9), 140 (4), 139 (17), 138 (11), 129 (13), 128 (12), 127 (10), 126 (11), 125 (9), 119 (5), 118 (17), 113 (32), 112 (17), 111 (100), 110 (78), 105 (23), 104 (15), 103 (12), 102 (13), 100 (6), 99 (16), 92 (8), 91 (30), 87 (3), 78 (11), 77 (73), 76 (31), 75 (37), 66 (4), 65 (23), 64 (17), 63 (24), 62 (9), 61 (14), 60 (10), 52 (9), 51 (42), 50 (26); Anal. Calcd. for C_18_H_15_ClN_6_S (382.87): C, 56.47; H, 3.95; Cl, 9.26; N, 21.95; S, 8.37. Found: C, 56.66; H, 3.83; Cl, 9.40; N, 22.04; S, 8.54.

*2-(4-Chlorophenylhydrazono)-2-(5-ethylthio-4-phenyl-4H-[1,2,4]triazol-3-yl)acetonitrile* (**8B**). Reddish crystals. Yield (0.60 g, 38%); m.p.: 219–221 °C. IR (KBr): *v* = 3240 (NH), 2924 (aliph. CH), 2220 (CN), 1595 (C=N), 1233 (N-N) cm^−1^; ^1^H-NMR: δ = 1.44 (t, 3H, *J* = 7.2 Hz, CH_3_), 1.48 (t, 3H, *J* = 7.2 Hz, CH_3_), 3.28–3.37 (m, 4H, 2 CH_2_), 6.29 (d, 2H, *J* = 9 Hz, ArH), 7.10 (d, 2H, *J* = 9 Hz, ArH), 7.31–7.38 (m, 8H, ArH), 7.57–7.69 (m, 6H, ArH), 8.79 (s, 0.17H, hydrazone NH), 13.74 (s, 0.83H, triazole NH); ^13^C-NMR: δ = 14.69 (2 CH_3_), 26.65 (CH_2_), 26.78 (CH_2_), 99.71 (=C-CN in azo-enamine form), 114.05 (CN), 115.45 (CN), 116.68 (3 Ar-C), 127.36 (1 Ar-C), 128.20 (4 Ar-C), 129.02 (1 Ar-C), 129.34 (1 Ar-C), 129.65 (4 Ar-C), 129.87 (1 Ar-C), 129.93 (1 Ar-C), 130.16 (4 Ar-C), 131.52 (1 Ar-C), 131.64 (2 Ar-C), 139.44 (1 Ar-C), 140.47 (CN-C=N-NH in hydrazone form), 148.30 (triazole C-3), 148.52 (triazole C-3), 154.40 (triazole C-5), 155.05 (triazole C-5); MS *m/z* (rel. int. %) 384 (M^+^, 10), 382 (M^+^, 29), 381 (11), 362 (3), 356 (3), 355 (3), 354 (5), 353 (4), 352 (2), 348 (3), 328 (3), 327 (4), 326 (4), 325 (7), 324 (2), 319 (6), 317 (8), 316 (5), 293 (3), 244 (2), 243 (3), 242 (7), 241 (3), 231 (2), 215 (7), 214 (4), 213 (2), 157 (4), 156 (9), 155 (6), 149 (4), 143 (2), 142 (4), 141 (9), 140 (4), 139 (29), 129 (5), 128 (8), 127 (5), 126 (12), 125 (7), 119 (2), 118 (6), 113 (31), 112 (20), 111 (100), 110 (27), 105 (8), 104 (4), 103 (4), 102 (5), 101 (8), 100 (3), 99 (17), 91 (13), 90 (12), 87 (3), 78 (4), 77 (31), 76 (17), 75 (36), 74 (13), 66 (2), 65 (9), 64 (12), 63 (17), 62 (6), 61 (7), 60 (4), 52 (5), 51 (23), 50 (17); Anal. Calcd. for C_18_H_15_ClN_6_S (382.87): C, 56.47; H, 3.95; Cl, 9.26; N, 21.95; S, 8.37. Found: C, 56.61; H, 4.04; Cl, 9.33; N, 21.87; S, 8.48.

## 4. Conclusions

In conclusion, we have synthesized new azo dyes utilizing 3-ethylthio-5-cyanomethyl-4-phenyl-1,2,4-triazole as a coupling component. The experimental results show that the substituents at the *para*-position of the diazonium salt benzene ring have some effect on the ratio of the resulting tautomers. 5-ethylthio-*N****'***,4-diphenyl-4*H*-1,2,4-triazole-3-carbohydrazonoyl cyanide (**3B**) (hydrazone form) was obtained by coupling **1** with benzenediazonium salt **2**, while the azo dye **5A** was obtained by coupling **1** with diazotized 4-methylaniline (**4**). Interestingly, coupling of **1** with diazotized 4-chloroaniline (**6**) afforded two isomeric products, **7A** (azo form) and **8B** (hydrazone form). To the best of our knowledge, this is the first reported isolation of two isomers in the solid state in such reactions. Analysis of the ^1^H-NMR data shows that the hydrazone and azo-enamine forms are the only two tautomers present in CDCl_3_ solution and the ratio of these tautomers depends on the electron-donating and electron-withdrawing properties of the substituent present at the *para-*position of the aryldiazonium salt.
